# A Polyvinylpyrrolidone Nanofibrous Sensor Doubly Decorated with Mesoporous Graphene to Selectively Detect Acetic Acid Vapors

**DOI:** 10.3390/s24072174

**Published:** 2024-03-28

**Authors:** Paolo Papa, Emiliano Zampetti, Fabricio Nicolas Molinari, Fabrizio De Cesare, Corrado Di Natale, Giovanna Tranfo, Antonella Macagnano

**Affiliations:** 1 Institute of Atmospheric Pollution Research (IIA)-CNR, 00010 Montelibretti, RM, Italy; emiliano.zampetti@cnr.it (E.Z.); or fmolinari@inti.gob.ar (F.N.M.); decesare@unitus.it (F.D.C.); 2 National Institute of Industrial Technology (INTI), Buenos Aires B1650WAB, Argentina; 3 Department for Innovation in Biological, Agro-food and Forest Systems (DIBAF), University of Tuscia, 01100 Viterbo, VT, Italy; 4 Department of Electronic Engineering (EED), University of Tor Vergata, 00133 Rome, RM, Italy; dinatale@uniroma2.it; 5 Department of Medicine, Epidemiology, Occupational and Environmental Hygiene (DIMEILA)-INAIL, 00144 Monteporzio Catone, RM, Italy; g.tranfo@inail.it

**Keywords:** acetic acid detection, electrospun nanocomposite nanofibers, mesoporous graphene, selective sensor, portable sensing tool

## Abstract

An original approach has been proposed for designing a nanofibrous (NF) layer using UV-cured polyvinylpyrrolidone (PVP) as a matrix, incorporating mesoporous graphene carbon (MGC) nanopowder both inside and outside the fibers, creating a sandwich-like structure. This architecture is intended to selectively adsorb and detect acetic acid vapors, which are known to cause health issues in exposed workers. The nanocomposite MGC-PVP-NFs layer was fabricated through electrospinning deposition onto interdigitated microelectrodes (IDEs) and stabilized under UV–light irradiation. To enhance the adhesion of MGC onto the surface of the nanocomposite polymeric fibers, the layer was dipped in a suspension of polyethyleneimine (PEI) and MGC. The resulting structure demonstrated promising electrical and sensing properties, including rapid responses, high sensitivity, good linearity, reversibility, repeatability, and selectivity towards acetic acid vapors. Initial testing was conducted in a laboratory using a bench electrometer, followed by validation in a portable sensing device based on consumer electronic components (by ARDUINO^®^). This portable system was designed to provide a compact, cost-effective solution with high sensing capabilities. Under room temperature and ambient air conditions, both laboratory and portable tests exhibited favorable linear responses, with detection limits of 0.16 and 1 ppm, respectively.

## 1. Introduction

Currently, polymer nanocomposites represent one of the most significant areas of focus in polymer chemistry and nanotechnology research, including coating and printing [1,2,3], smart packaging [4], advanced electronics [5], energy storage and conversion devices [6], biomedical tools [7], drug delivery vehicles, antimicrobial materials [8,9], and sensor technology [10]. Polymer nanocomposites have sparked considerable interest in sensor applications over the past few decades, emerging as pivotal components in sensor system design. Hence, given the demand for innovation in sensors and the transition from traditional to high-performance, portable, and nanoscale systems, the scientific community has extensively investigated the development of advanced combinations of nanomaterials and polymer matrices for sensor applications. This interest is largely aroused by the multitude of advantages these materials offer, such as their outstanding electrical and mechanical properties, sensitivity, and simplicity of manufacturing [11]. Actually, the exceptional blend of versatility, processability, functionalization, biocompatibility, scalability, and customized properties renders nanocomposite polymers highly appealing for sensor nanotechnology, facilitating diverse applications. These sensors can be synthesized on a large scale through cost-effective methods, rendering them economically feasible for mass production. Additionally, they can be imbued with a diverse array of functional groups, additives, or nanoparticles to confer specific properties, such as conductivity, biocompatibility, or responsiveness to distinct external stimuli.

In this study, a selective sensor for acetic acid vapors based on the combination of non-conductive polymer nanofibers (polyvinylpyrrolidone, PVP) with nanopowders of mesoporous graphitized carbon (MGC) is described. As far as authors know, there is limited literature on specific acetic acid vapor sensors [12,13,14,15,16,17,18,19,20,21], and they are not commonly found among commercially available sensor options [22,23,24,25]. So far, the rarity of commercial sensors specifically designed to detect acetic acid vapors (for instance, based on electrochemical cells [26] or colorimetric sensing technology [27]) could be attributed to several factors. The use of acetic acid sensors is often limited to specific industries or applications where the detection of acetic acid vapors is critical, such as in chemical manufacturing (e.g., paints, adhesives, plastics, and textile finishes, cleaners), food processing (e.g., flavoring, preservation, acidification, and sanitizing), or pharmaceutical production (e.g., solvent in synthesis, pH adjustment, drug delivery systems, and excipient in formulations). This specialization restricts their widespread adoption compared to sensors for more commonly monitored gases. Additionally, detecting acetic acid vapors accurately and selectively can be technically challenging due to factors such as interference from other gases, VOC cross-reactivity, variability in concentration levels, and the need for high sensitivity and specificity. Overall, while acetic acid sensors play a vital role in certain niche applications, their limited demand, technical challenges, cost considerations, and regulatory factors may contribute to their specialized status compared to sensors for more commonly monitored gases.

Additionally, acetic acid is considered an environmentally friendly chemical since it is biodegradable and produced from renewable sources according to sustainable routes [28]. On the other hand, its vapors are hazardous, with potential health risks (respiratory irritation, eye irritation, skin burns, and other health problems) overall for people exposed to high concentrations over prolonged periods.

Occupational exposure to acetic acid can occur through inhalation, skin contact, or eye contact. Acetic acid is corrosive to the skin and eyes, and the Occupational Safety and Health Administration (OSHA) has established standards for exposure to it. In Europe, the indicative occupational exposure limit value (IOELVs, from Commission Directive 2017/164/EU) for acetic acid is 10 ppm (25 mg/m^3^) over an 8 h work shift, and the short-term exposure limit (STEL) is 20 ppm (50 mg/m^3^) [29]. The main symptoms of exposure to acetic acid vapors at this level may include irritation of the eyes, nose, and throat. At concentrations of 100 ppm, individuals may experience significant lung irritation and possible damage to the lungs, eyes, and skin. Exposure to acetic acid can also lead to pharyngeal edema and chronic bronchitis.

Therefore, wearable sensors for acetic acid gas could play a vital role in protecting the health and safety of workers, ensuring regulatory compliance, and enhancing process efficiency and safety in industries where acetic acid is used.

In the last decade of literature, most of the planned and investigated sensors to monitor acetic acid used nanostructured metal oxide and their combinations for chemiresistors, which required high temperatures (ranging between 150 and 380 °C) to achieve high sensing performances (data listed in Table 1, Section 3.2) [13]. Their LODs varied between 10 ppb and 50 ppm, depending on the quality of doping and nanoarchitecture. Some dopants were selected for their catalytic properties; others were selected for their ability to create finer microstructures or grain boundaries, thereby increasing the surface area available for interaction with acetic acid molecules. For instance, electrospinning technology was employed to enhance the sensitivity of In_2_O_3_ for detecting HAc. The resulting highly porous and interconnected structure enabled the detection of the analyte at a concentration of 500 ppb when the sensor operated at 250 °C [21]. Conversely, ZnO was explored as a promising compound for detecting acetic acid at both high and room temperatures. The sensitivity of ZnO varied depending on whether it was in the form of hexagonal nanocrystals or foam surfactant [16], highlighting that an increase in the density of surface defects and active sites within a nanoarchitecture enhanced interactions with the analyte. By the way, the foam variant achieved an LOD of 500 ppb at a working temperature of 400 °C. The integration of a porous metal–organic framework (Tb_2_O_3_@MOF) [17] with ZnO enabled the sensor to operate effectively at room temperature. However, to enhance sensor sensitivity and achieve an LOD of 500 ppb, UV light excitation was employed. Conversely, sensors based on GQDs–ZnO composites (GQDs: graphene quantum dots) could be operated at room temperature and exhibited a stronger response to acetic acid gas compared to a pure ZnO sensor, detecting up to 1 ppm at room temperature [30]. The mesoporosity of a metal oxide (CuO) was utilized to create a sensor operating at 200 °C [31], whereas the incorporation of graphene (RGO or G) in conjunction with metals [32] or ceramics [33] enabled chemiresistors to function at room temperature with exceptional sensitivity (achieving a limit of detection of up to 1 ppb [34]). Avossa et al. (2018) reported that a chemiresistor based on ES nanofibers of a blend of polystyrene and polyhydroxybutyrate (PS-PHB) hosting MGC (0.93% mass ratio) was sensitive and selective to acetic acid vapors, but only worked at a temperature slightly higher than room temperature (T = 40 °C). The mesoporous structure, with a 137 Å average pore diameter, acted as a nucleation center for entrapping and growing acetic acid. Since the sensor did not appear to reach a plateau quickly, an LOD was not reported [35]. The necessity of the sensor to work at more elevated temperatures appeared to be related to the polymer’s structure and the heterogeneous network architecture of MGC within the fibers. By changing the hosting polymer, the electrical and sensing features could be improved. Thus, in the present study, the mesoporous graphene nanopowder was dispersed in a PVP solution to obtain nanocomposite nanofibers by electrospinning (PVP-MGC NFs). PVP looks like a popular choice for electrospinning technology [36] due to the combination of a series of features like excellent solubility in a wide range of solvents, including aqueous eco-friendly solvents, good electro-spinnability due to the formation of a stable jet under the influence of an electric field, blend compatibility, biocompatibility, and non-toxicity, allowing for the development of diversified nanofibers with enhanced mechanical and electrical properties, and cost-effectiveness. On the other hand, PVP;s solubility in water also renders it a delicate material for sensing layers. Exposing it to UV–light irradiation for a brief period generates radicals, leading to the formation of new bonds between the chains within the individual fibers. This process makes the fiber insoluble or alters its solubility in various solvents, imparting new chemical and physical properties, along with enhanced stability, that are contingent upon the duration of UV–light exposure [37]. The use of mesoporous graphene as a nanofiller is of significant interest in sensor applications [38] due to the combination of the excellent conductivity of graphene with a network of periodic mesopores, increasing the available surface area for interactions with surrounding molecules and electrons. More specifically, MGC consists of a single layer of sp^2^ carbon atoms bonded in a hexagonal honeycomb crystalline arrangement with exceptional physical properties, including high carrier mobility (up to 350·10^3^ cm^2^/(Vs)), thermal stability (as demonstrated by Bolotin et al., 2008 [39]), and high mechanical strength (with a Young’s modulus of 1 TPa and fracture strength of 130 GPa), and it is also then able to impart both conductivity and increased mechanical strength to the nanofibers. Additionally, the mesoporous structure, providing a larger available surface area (50–100 m^2^/g) to the nanopowder, offers the potential for enhanced selectivity through molecular size exclusion effects (with an average pore diameter of 137 Å). This material, pristine or oxidized and in combination with other compounds, has primarily found applications in the energy sector, catalysis, selective gas adsorption [40], and in bio- and chemical sensing [41,42,43,44].

Electrospinning technology is a versatile and scalable technique for producing nanocomposite nanofibers with controlled morphology and composition, offering a versatile and effective approach for designing wearable sensors with enhanced sensitivity, selectivity, flexibility, and biocompatibility, making it a promising strategy for various applications in healthcare, environmental monitoring, and beyond [45,46,47,48].

The versatility of electrospinning technology allows for the creation of advanced and sophisticated sensing layers that are compatible with electronics and electronic nanodevices [49]. Electrospun nanofibers have an exceptionally high surface area-to-volume ratio, which provides a large interface for interactions with target analytes. This increased surface area is expected to enhance the sensitivity of sensors by maximizing the number of active sites available for molecular adsorption and detection. The deposition process allows for precise control over the morphology, structure, and composition of nanofibers, enabling the design of sensing layers with specific properties tailored to the requirements of the target analyte and sensing application. The resulting nanofibrous sensing layers are inherently flexible and can conform to a plethora of surfaces, making them suitable for integration into wearable and conformable sensor devices. Further, electrospinning is a scalable manufacturing technique that can produce nanofibrous sensing layers over large areas and volumes. This scalability is essential for the commercialization and widespread adoption of sensing technologies in various applications and industries.

The electrospinning technique involves applying a high voltage, in the range of several kilovolts, between the spinneret of a spinnable polymer solution and a collector equipped with the transducer. The collected nanofibers may undergo additional processing steps, such as drying or crosslinking, to improve their mechanical properties and stability. The choice of polymer solution (i.e., based on homopolymers, blends, nanocomposites, metal oxide precursors, etc.) depends on the desired properties of the nanofibers and the intended application of the sensing electrospun materials [50].

It has been proven that the distribution of mesoporous graphene (MGC) within the electrospun polymer nanofibers [51,52,53,54,55] depends on factors such as the graphene/polymer mass ratio, the affinity to the hosting polymers, and the parameters of the electrospinning process. Storti et al. (2023) [56] successfully embedded graphene nanoplatelets into PVP nanofibers without further additives, achieving homogeneous distributions through pulsed ultrasonication and testing as an antimicrobial tool. Similarly, Del Sorbo et al. (2019) [57] developed nanocomposite fibers of PVP and non-covalently functionalized graphene through tip sonication of graphene alcoholic suspensions in the presence of PVP and used them as sound absorbing-materials.

In this study, the architecture of PVP-MGC nanofibers was developed over a commercial interdigitated electrode (IDE) to function as a sensor for the rapid and selective detection of acetic acid vapors, operating within the previously mentioned permissible exposure limits (PELs) established for acetic acid [28] and serving as a promising candidate for integration into portable monitoring systems aimed at protecting workers.

The selection of UV-cured PVP nanofibers as the matrix aims to ensure the long-term stability and performance of the sensor device, even under harsh operating conditions.

To enhance both electrical conductivity and sensing features, the outer layer of the nanocomposite nanofibers was additionally decorated with MGC nanopowder, serving the dual roles of filler and surface binding agent. The mesoporous structure of MGC should provide abundant active sites for analyte adsorption, leading to enhanced sensing sensitivity. Additionally, the high conductivity of graphene is expected to facilitate efficient electron transfer, resulting in rapid and responsive sensing performance. The double decoration of PVP nanofibers with MGC nanopowder aims to synergistically enhance the sensor’s sensitivity, selectivity, and stability for detecting acetic acid vapors.

Preliminary measurements were carried out by connecting the sensor to an electrometer to detect changes in current.

Subsequently, the sensor was incorporated into a portable sensing device using the Arduino architecture system. It was then subjected to analyte detection tests for a few tens of seconds, covering the concentration ranges of interest. This approach is in line with the objective of developing a portable system capable of providing a cost-effective and transportable solution with advanced sensing capabilities for use in workplace risk scenarios.

## 2. Materials and Methods

### 2.1. Materials

All the materials and chemicals used in this work were of analytical grade and used as received. Mesoporous graphitized carbon nanopowder (MGC, >99.95%, <500 nm DLS, 50–100 m^2^/g, <200 nm particle size), polyvinylpyrrolidone (PVP, Mw~1,300,000), ethanol anhydrous (EtOH 99,8%), polyethyleneimine (PEI, Mw~423), acetic acid (HAc, ≥99%), formic acid (FA, ≥98%), methanol (MeOH, ≥99.8%), acetone (Ac, ≥99.5%), triethylamine (TEA, ≥99.5%), and butylamine (ButA, ≥99.5%) were purchased from Merck KGaA (Darmstadt, Germany).

Interdigitated electrodes (IDEs), supplied by Micrux Technologies (Gijón, Spain), were fabricated on glass substrates (IDE dimensions: 10 × 6 × 0.75 mm, Pt/Ti electrodes, 120 pairs, 10 μm wide × 5 mm long × 150 nm thick, with a 10 μm gap). Prior to use, the electrodes were cleaned with a soap solution and a “base piranha” mixture at 60 °C for approximately 30 min (3:1, v:v, ammonia water, and hydrogen peroxide water solution), followed by rinsing with Milli-Q water (~18 MΩ cm).

### 2.2. Sensor Material Growth

The MGC suspension, prepared by subjecting it to pulsed ultrasonication (10 min, Branson 1800) followed by alternating vortexing and magnetic stirring until a dark ink-like liquid was achieved, was combined with the PVP/EtOH solution (C: 58.2 mg/mL). The mixture was blended until a PVP:MGC solution ratio of 1:0.0045 (w:w) was obtained for electrospinning and loaded into a syringe placed inside the electrospinning deposition chamber.

**Figure 1:**
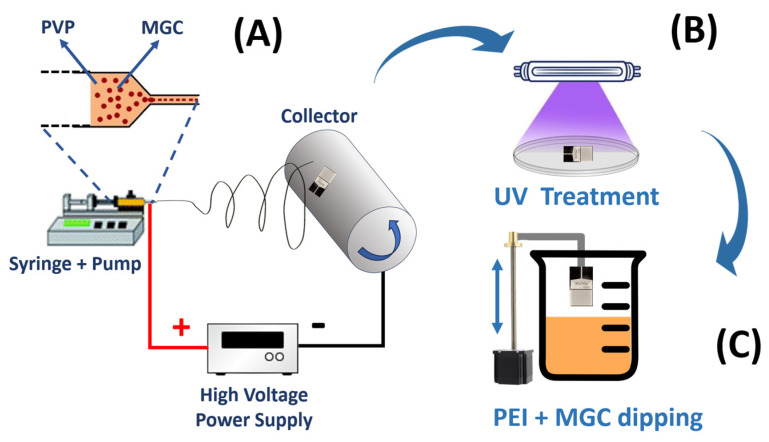
Scheme of the electrospinning deposition process (**A**), UV–light curing of the PVP-MGC nanofibrous sensing layer, (**B**) and MGC-PEI coating of NFs by dipping (**C**).

The fiber deposition process was carried out using a Fluidnatek^®^ LE-50 electrospinning machine (Bioinicia, Paterna, Valencia, Spain). To ensure the production of uniform and dry fibers, the distance between the needle and the collector was set at 9 cm, with a solution flow rate of 400 µL/h. In the electrospinning setup, the needle was charged to a voltage of +4.6 kV, while the collector maintained a potential of −2 kV (Figure S1). A rotating drum collector (500 rpm) was applied to promote a more organized alignment of the fibers during deposition. Once the electric potential was applied, the polymeric dispersion jet coated the interdigitated electrodes (IDEs) secured to the collector using conductive tape and positioned inside the deposition cone; see Figure 1A.

The UV–light photocrosslinking of the polymer composite nanofibers occurred using a 500 W UV lamp (Polimer Helios Italquartz, Cambiago, MI, Italy) emitting in a spectrum range from 180 nm to visible light for 10 min. Samples were placed 10 cm away from the light source, with the film temperature set to 28 °C by a Peltier cell; see Figure 1B.

The dipping suspension contained polyethyleneimine (PEI, C: 10.7 mg/mL) and MGC (C: 0.4 mg/mL) in EtOH, previously subjected to pulsed ultrasonication, vortexing, and magnetic stirring.

The deposition by dipping occurred using a custom-built system, enabling precise control and regulation of both immersion and withdrawal speeds set at 1 mm per second; see Figure 1C.

A scheme of the entire deposition process and sensor development is represented in Figure 1.

### 2.3. Material Characterization

The scanning electron microscopy (SEM) technique was employed to characterize the size, shape, architecture, and surface properties of the nanofibers. Specifically, nanofibrous fabrics produced via electrospinning were deposited onto thin SiO_2_ wafers and gold sputter-coated using a Balzers MED 010 unit. These samples were then analyzed using a JEOL JSM 6010LA electron microscope at the High Equipment Centre, University of Tuscia, Viterbo, Italy.

The average diameter of the fibers was determined using Gwyddion© 2.64 software (GNU General Public License), with measurements conducted on a total of 150 fibers per sample. The normal probability distribution was calculated based on the respective means and standard deviations using Microsoft^®^ Excel^®^ (Microsoft 365 MSO, version N. 2402).

Quality assessment of fibers on the IDE surface was conducted utilizing an optical microscope, specifically the Leica DM2700M (Leica Microsystems CMS GmbH, Wetzlar, Germany), with observations made at magnifications of 20×, 50×, and 100×. The images were captured by Leica DMC4500 camera under incident light in brightfield using the LED lamp LH113, as well as in fluorescence using the Lamp EL6000 with FITC-Texas filters for green and blue excitation, respectively.

Transmission electron microscopy (TEM) micrographs were acquired at 200 keV using a transmission electron microscope equipped with an analytical double-tilt probe. Electrospun nanofibers were collected in static mode on nickel grids (Gilder Grids, 50 mesh, 3.05 mm O.D., Nickel) for a few seconds and observed without any fixative or staining using a JEOL 1200EXII electron microscope (JEOL, Peabody, MA, USA). Micrographs were captured using an SIS VELETA CCD camera (Olympus, Muenster, Germany) equipped with iTEM software (TEM Imaging Platform).

### 2.4. Sensor Measurement Systems Setup

**Figure 2:**
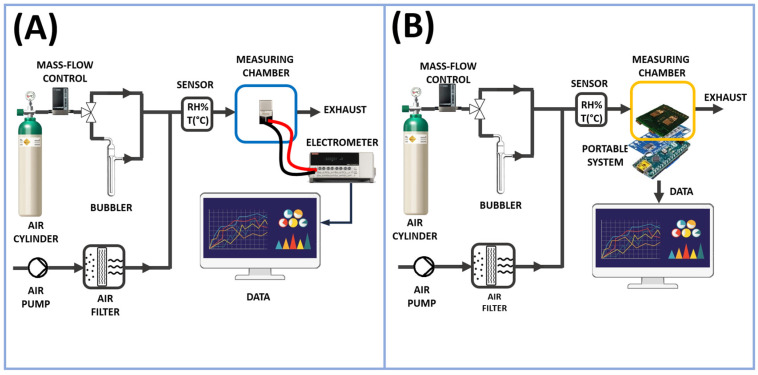
Layout of the measurement tests performed with the stationary (**A**) and the portable (**B**) system.

The measurements presented in this study were initially conducted on a laboratory bench system and later replicated (in the same measurement conditions) using a low-cost portable measurement device. The two-system setup is depicted in Figure 2A,B.

As previously mentioned, the vapor tests were performed with the stationary measurement system; see Figure 2A. In this way, the resulting chemiresistor (consisting of IDEs and NFs, where NFs refers to nanofibers) was enclosed in a measuring chamber with a volume of approximately 3 mL, and then linked to an electrometer (Keithley 6517, Solon, OH, USA) capable of both powering and measuring its electrical outputs, with data transmission to a PC facilitated by LabVIEW Software (2014) from National Instruments (Austin, TX, USA). The current, recorded under clean air conditions, was monitored by applying potential values ranging from 0 to 2.0 V in increments of 0.2 V, with humidity percentages and temperature values strictly controlled. The resistance (R) of the fibrous coating and its relationship to humidity at 25 °C (45% RH) were determined using Ohm’s Law, which states that the resistance of a circuit equals the voltage across it divided by the current flowing through it. VOC measurements were carried out by applying 1 volt of potential. The measurement chamber was conditioned by a customized pneumatic system necessary to generate the desired vapor concentrations. It comprised a mass flow control (MFC) with a range capacity of 0–200 standard cubic centimeters per minute (sccm) managed by a 4-channel readout controller (MKS Type 247 from MKS Instruments, Andover, UK), an electrovalve to switch the fluxes (S070C-RAG-32 from SMC Corporation, Tokyo, Japan), and an air pump (NMP015, KNF, Freiburg im Breisgau, Germany) to generate a carrier ambient air flux, which was previously purified, passing through a carbon-activated cartridge. All the connections between Teflon tubes were guaranteed by Festo (Festo AG & Co. KG, Esslingen, Germany) push-in connectors. An air cylinder (5.0, Nippon Gas Italia S.r.L., Milan, Italy) was used as a carrier gas in the circuit dedicated to the bubbling and collection of VOC vapors. This configuration facilitated the generation of precisely controlled concentrations of volatile organic compounds (VOCs). The concentration range for acetic acid was determined based on its application range (0.95–30 ppm), while for all other VOCs, the concentration range was selected to ensure it reached at least a value detectable by the sensor (FA: 5–440 ppm, NH_3_: 7–755 ppm, MeOH: 14–2245 ppm, EtOH: 7–930 ppm, Ac: 25–4125 ppm, TEA: 17–1350 ppm, and ButA: 16–1690 ppm).

**Figure 3:**
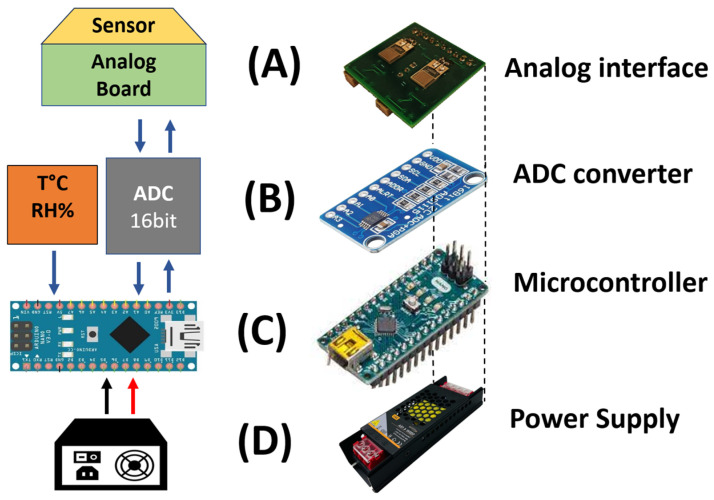
Representation of the portable measurement system with a layered structure in analogue board (**A**), ADC module (**B**), microcontroller (**C**), and the power supply (**D**).

The second measuring setup (Figure 2B), similar on the pneumatic side, consisted of an electronic board (an analog board) connected to an IDE that converted the electric resistance variations into voltage variations; see Figure 3A. A 16-bit analog to digital convert (ADC) module (ADS1115 by Analog Devices, Wilmington, MA, USA), Figure 3B, converted the analog signals into digital ones. A microcontroller (Arduino Nano, by Arduino SA, Chiasso, Switzerland), Figure 2B, acquired and transmitted the generated data to a computer unit through a USB port. A software program (developed by Labview) elaborated, plotted, and stored these data in real-time. The software executed measurement cycles and generated text files (.txt or .csv).

In this way, the system could register the presence of possible threshold levels and provide the possibility to subsequently analyze the stored data, giving a long-term average concentration to which the operator was exposed.

Finally, in order to operate, the entire portable system required a power supply (5 V DC) that was connected to the mains power. It was located at the bottom of the system, as depicted in Figure 3D.

## 3. Results and Discussion

### 3.1. Sensing Material Characterization

Electrospinning technology facilitated the one-step creation of nanocomposite nanofibrous layers using a single needle. Depositions were easily performed on various substrates, including silicon dioxide thin slices for morphological and optical characterization of the fibers and customized borosilicate interdigitated electrode (IDE) transducers for measuring the electrical and sensing properties of the thin nanofibrous coating.

Each substrate, securely fixed onto the grounded rotating cylinder and aligned with the needle tip, efficiently collected the ejected fibers. The electrospun jet streams maintained uninterrupted flow, leading to the formation of a fibrous network within a mere four minutes. The exposure of PVP nanofibers to UV light irradiation was expected to initiate the photocrosslinking of the polymer in the solid state, aided by the production of O_3_ radicals in the surrounding air.

PVP, being water-soluble and also soluble in ethanol and most polar solvents, is inherently fragile and not ideal for sensing applications. Previous studies have demonstrated that UV irradiation can form insoluble and photocrosslinked PVP structures [58]. Depending on the duration of exposure, these bonds made the PVP fibers insoluble or differently soluble in various solvents, endowing them with altered chemical and physical properties and increased stability.

Initially, PVP nanofibers were grown using identical electrospun deposition parameters for both versions, with and without the inclusion of MGC (Figure S1). Due to its amphiphilic structure, comprised of a hydrophilic pyrrolidone moiety and hydrophobic alkyl groups, PVP has been commonly employed as a capping agent to enhance the stability and dispersibility of graphene and its derivatives in both aqueous and organic solvents. For instance, Wajid et al. reported the utilization of PVP as a stabilizing agent for dispersing pristine graphene at high concentrations across a wide range of organic solvents [59]. Thus, a good dispersion of MGC was expected.

Subsequently, the nanofibers were subjected to UV–light irradiation.

**Figure 4:**
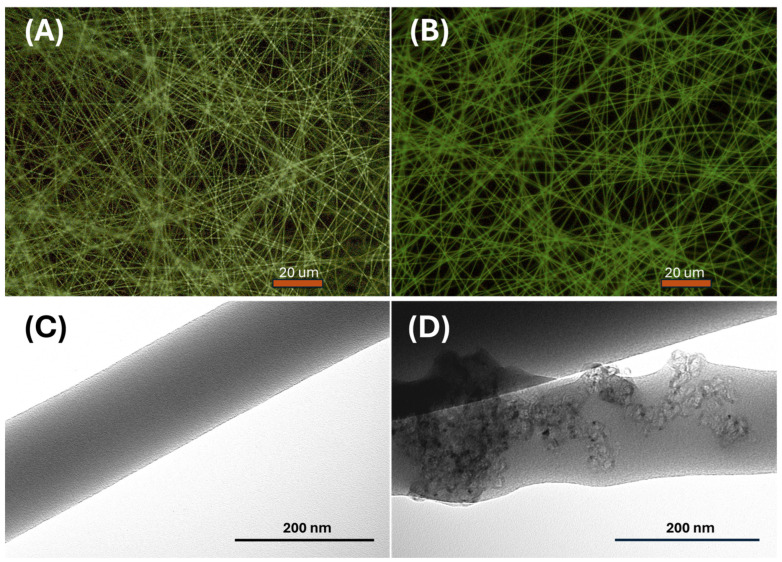
Fluorescent optical microscope views of PVP NFs (**A**) and PVP-MGC NFs (**B**) collected on SiO_2_ slice; TEM pictures of PVP (**C**) and PVP-MGC (**D**) nanofibers.

These fibers exhibited a uniform structure without any apparent beads or globular formations, as evidenced by the fluorescence optical microscopy images depicted in Figure 4A,B. While PVP is not typically recognized as a fluorescent polymer, it demonstrates significant intrinsic fluorescence, particularly under conditions of photo-oxidation [60,61]. The distinguishing feature between the two fiber types is highlighted by variations in fluorescence emission intensity and brightness. This effect could be attributed to a combination of factors, including enhanced light absorption by the mesoporous graphitized carbon, promoting a more efficient excitation of fluorescence within the nanofibers. Additionally, synergistic effects between materials could occur, such as charge transfer phenomena, thus contributing to changes in the optical properties of the nanofibers.

Conversely, transmission electron microscopy (TEM) images reveal distinct characteristics between the two types of polyvinylpyrrolidone (PVP) nanofibers (Figure 4C,D). The first type exhibits a smooth and regular morphology, with uniform diameter and surface texture, as observed in the TEM image in Figure 4C. In contrast, the second type of PVP contains bunches of MGC along the fiber structure, resulting in localized humps or slight irregularities in the external shape of the fiber. The PVP-MGC fibers served as the scaffold for the conductometric sensor designed to detect acetic acid.

**Figure 5:**
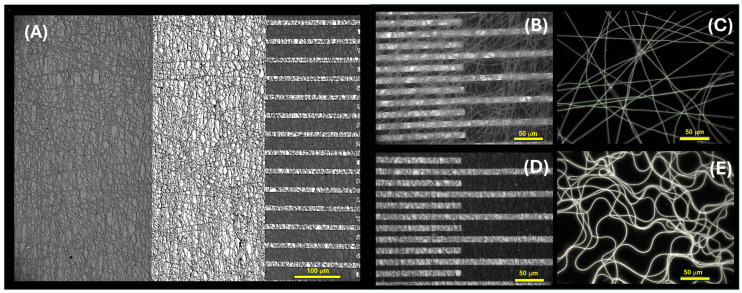
Optical microscope images depict the distribution of fibers on the IDE surface in brightfield (**A**,**B**,**D**) and SiO_2_ wafer slices under fluorescence (**C**,**E**). Specifically: (**A**,**D**) show the (PVP-MGC)^UV^/PEI-MGC nanofibers coating the electrodes following a 4-min electrospinning deposition; (**B**) shows the (PVP-MGC)^UV^ electrode nanofibrous coating before dipping; (**C**) displays the (PVP-MGC)^UV^ nanofibrous layer from a 30-s deposition; and (**E**) illustrates the layer following the decoration with PEI-MGC.

As reported in Figure 5A, the interdigitated electrodes with electrospun nanofibers exhibited uniform coverage, forming a network architecture characterized by consistent microporosity. These pores were supposed to serve as pathways for gas transport, thereby bolstering the material’s suitability for gas/VOC sensing applications.

**Figure 6:**
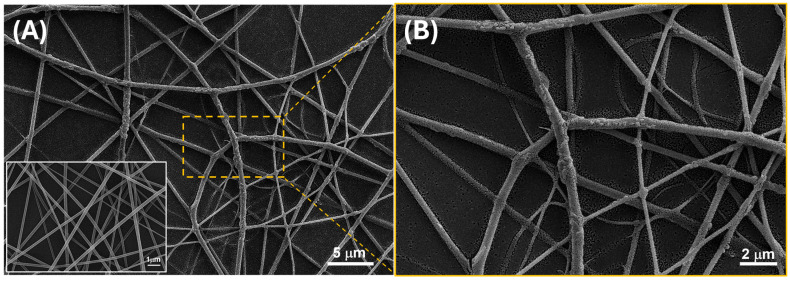
SEM micrographs depict fibers of PVP-MGC after UV–light irradiation (A-*inset*) and subsequent PEI-MGC decoration through dipping (**A**), along with a magnified view of a section (**B**).

Optical microscope pictures of Figure 5B,C and SEM micrographs of Figure 6 (A-*inset*) highlight a partially aligned orientation of nanofibers as deposited and subjected to UV-irradiation, suggesting a certain degree of directional organization of the material. The following dipping of the layer into a solution of PEI-MGC altered the shape distribution of the fibers, leading to bundling, branching, and undulations in the fibers, as observable in both Figure 5A,D,E and Figure 6A,B. This effect may happen for the interactions between EtOH and the polymer chains and be responsible for a partial swelling and then disruption of the linear arrangement. Such a fibers shape change is highlighted by fluorescence microscope pictures (Figure 5C,E) of a looser mesh network before and after dipping. Indeed, the fibers exhibited a notable brightness, leaning towards a green hue (Figure 5C).

Moreover, when coated with PEI oligomers and MGC nanopowder, PVP nanofiber luminosity intensified further, accompanied by a shift in emission. Under the fluorescent optical microscope, the presence of a thin, radiant coating on the fibers was clearly observable (Figure 5E) due to the bright blue fluorescence of PEI [62] confirming the dipping deposition.

However, following the dipping, analysis through both optical and scanning electron microscopy revealed that the fibers collected in denser networks (Figure 5A,D and Figure 6A,B exhibited enhanced adhesion to the substrate (IDEs and SiO_2_ wafer, respectively) and maintained interconnectivity with one another, despite experiencing partial loss of their linear structure.

SEM images of Figure 6 (A-*inset*) proved that the individual nanofibers within the network exhibited intersecting trajectories, creating points of contact and potential bonding between adjacent fibers.

These intersections contribute to the formation of junctions, thereby enhancing the structural integrity of the three-dimensional nanofiber network.

The increased complexity of the network is evident in the emergence of features such as junctions, cross-linkages, and overlapping segments of nanofibers.

These characteristics are expected to bolster the overall stability and mechanical strength of the three-dimensional structures, while also increasing the available surface area with adsorption sites.

**Figure 7:**
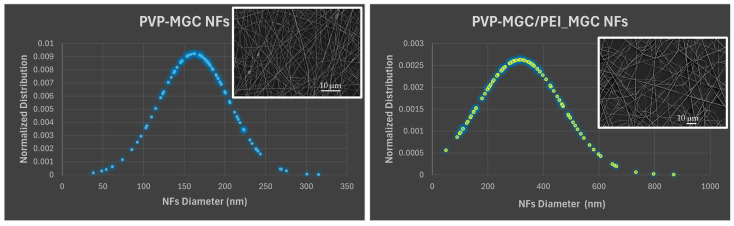
Normalized distribution of NF diameters of PVP-MGC (**left**) and PVP-MGC/PEI-MGC (**right**). The *x*-axis represents the range of nanofiber diameters; the *y*-axis represents the probability density of nanofibers at each diameter.

The normalized distribution of the fiber dimension is reported in Figure 7. The graphs show that prior to coating, the nanofibers exhibited a relatively narrow and well-defined distribution of diameters, as depicted by the Gaussian curve in Figure 7 (left), with a mean diameter of 168.10 nm and an SD of 44.97 nm. However, after dip-coating with PEI-MGC, the average diameter of the nanofibers increased due to the addition of the material onto the surface of the nanofibers (Figure 7, right). Both the flattening and broader shape of the Gaussian curve highlight a wider range of diameters within the coated nanofiber population (∅: 316.70 ± 151.85 nm). Indeed, during the dipping process, the fibers transitioned from smooth and uniform surfaces to surfaces adorned with rough sleeves. This transformation imparted a wrinkled appearance to the fibers and increased both their diameter (+188%) and heterogeneity in shape (Figure 6A,B). As a result, the nanofibers presented clusters of particulate matter distributed along their length with different surface densities but firmly adhering to the fiber due to the presence of PEI.

### 3.2. Sensing Electrical Characterization

**Figure 8:**
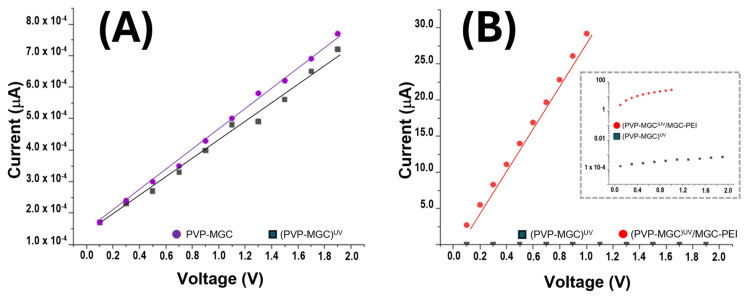
I/V measurement of the nanocomposite fibers before (PVP-MGC) and after the UV treatment (PVP-MGC)^UV^) (**A**); comparison between the I–V curves of (PVP-MGC)^UV^ and (PVP-MGC)^UV^/PEI-MGC (**B**).

Due to PVP nanofibers’ intrinsic poor electrical conductivity [63], the addition of MGC as a conductive filler is expected to enhance the overall electrical conductivity of the composite material. Figure 8A,B illustrates the current/voltage (I–V) plot for the IDE coated with PVP-MGC nanofibers before and after UV photocuring. The x-axis of the plot denotes the applied voltage across the electrode, ranging from 0 to 2 V, while the y-axis represents the current passing through the electrode. As the voltage is incrementally raised in the positive direction, both IDEs exhibit a linear increase in current, characterized by comparable slopes (≈2^3^ MOhm). A very slight increase in electrical resistance is observed when PVP-MGC nanofibers are UV-irradiated (PVP-MGC)^UV^, Figure 8A. It could be attributed to the alteration of the nanofiber structure induced by photooxidation/crosslinking, leading to changes in the conductivity pathways within the nanofiber network or in chemical/physical changes of the PVP-MGC interface.

However, the linear shape observed in the current/voltage curve of the PVP/mesoporous graphene nanofibers within the range of 0 V and +2 V suggests that MGC may be uniformly distributed inside the fibers and that the contact between fibers and electrodes implies that there is no significant energy barrier at the interface. This uniform distribution facilitates consistent electrical conductivity across the entire length and volume of the nanofibers. Further, it presumably indicates effective integration of the conductive material within the polymer matrix, ensuring efficient electron transport pathways. The integration of these nanofillers was also confirmed by the TEM images (Figure 4D). Graphene, being a highly conductive material, could introduce n-type doping characteristics to the composite nanofibers. The presence of defects or functional groups on the graphene surface may donate electrons to the PVP matrix, leading to an excess of negative charge carriers (electrons) and resulting in n-type semiconductor behavior. Interaction between the PVP polymer and graphene mesoporous structures may facilitate charge transfer processes.

The PEI-MGC decoration of fibers through dipping significantly boosted sensor conductivity (R ≈ 34 kOhm), while maintaining a linear relationship between the applied voltage and the measured current (Figure 8B). The inset graph in Figure 8B is identical to the one depicted in the same figure, except for the y-axis, which is presented in a logarithmic scale. This adjustment enables the visualization of IV curves simultaneously. Such an increase in current is presumably due to the outer MGC being able to provide additional conductive pathways with the nanofiber nanofiller network. PEI may further improve electrical conductivity by promoting better dispersion and adhesion of the mesoporous graphene onto the nanofiber matrix. Additionally, the decoration with mesoporous graphene and PEI could increase the surface area of the nanofibers, providing more active sites for electron transfer. This increased surface area is expected to facilitate a better interaction between the nanofibers and the surrounding environment, leading to enhanced sensing performance. Furthermore, PEI, known for its ability to promote charge carrier mobility [64], could contribute to the movement of electrons through the nanofiber network.

To evaluate the sensing features, we subjected both (PVP-MGC)^UV^ and (PVP-MGC)^UV^/MGC-PEI sensors to airflow under conditions of constant temperature and relative humidity. Each measure was carried out to detect various common solvents and chemical compounds that could potentially interfere with the sensors and might be commonly encountered in environments of laboratories and industries. For these measurements, defined amounts of fluxes were partialized and controlled to generate the necessary concentrations of the desired target vapors.

Upon exposure to each VOC, both the sensors demonstrated an increase in current. However, the responses of the (PVP-MGC)^UV^/MGC-PEI sensor were notably faster, as expected, and more reproducible than (PVP-MGC)^UV^, presumably attributable to the improved stability conferred by the addition of an outer skeleton of a mixture of nanopowder and oligomers, i.e., MGC and PEI, respectively.

For each of the VOCs tested, (PVP-MGC)^UV^/MGC-PEI sensor detected up to eight concentrations, starting from their saturated vapor pressure. From these measurements, variations in current corresponding to vapor concentrations were observed in the sensor output.

**Figure 9:**
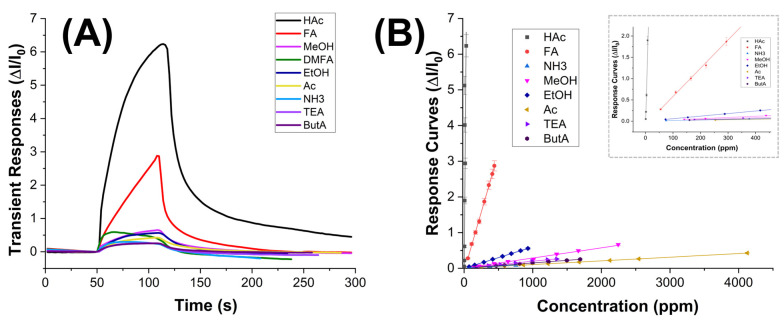
Normalized transient responses (ΔI/I_0_) towards the highest concentration of VOCs measured (HAc: 30 ppm, FA: 440 ppm, NH3: 754 ppm, MeOH: 2245, EtOH: 930 ppm, Ac: 4126 ppm, TEA: 1349 ppm, ButA: 1691 ppm) (**A**) and normalized response curves plotted against increasing concentrations (ppm) of VOCs (**B**).

The graph in Figure 9A depicts a comparison of the sensor’s transient responses upon exposure to different tested VOCs. These responses are calculated as the ratio between the changes in measurement current (ΔI) and the baseline current (I_0_) over time. Notably, for acetic acid (HAc) (at a concentration of 30 ppm), the current exhibited a rapid increase, stabilizing at t_90_ = 90 s, i.e., the time it takes for the sensor to reach 90% of its final stable response.

Conversely, the sensor did not reveal any signal to all the other VOCs at equivalent concentrations. However, it is noteworthy that these VOCs generate varying concentrations in parts per million (ppm) at room temperature due to differences in their partial pressures. Thus, the transient measurements in Figure 9A describe a comparison of responses among different concentration levels. In the case of formic acid (FA) that has a vapor pressure of 4.66 kPa (HAc, P_vap_: 1.54 kPa) calculated by Antoine Equation [65], the sensor demonstrated a distinct increase in current when exposed to approximately 440 ppm, although with slower kinetics and without reaching apparent equilibrium within the same exposure time defined vs. all the VOCs. Therefore, VOC molecules adsorbed onto the nanocomposite fibers, by changing the charge distribution, led to an increase in conductivity. However, despite being measured at concentrations ranging from hundreds to thousands of ppm, all other VOCs minimally influenced the current variation, as also confirmed in Figure 9B.

On the other hand, the sensor response size and shape to HAc suggested rapid and selective detection of the target analyte, which is essential in applications where real-time monitoring or quick identification of substances is required, such as environmental monitoring or industrial process control, where delays in sensor response could result in missed events or inaccurate readings. Moreover, the rapid response time correlated with the highest response signal enables the sensor to detect even low concentrations of analytes quickly. This is vital for ensuring the sensor’s effectiveness across a wide range of concentrations and for detecting trace amounts of acetic acid.

The graph depicted in Figure 9B showcases the correlation between normalized sensor responses and increasing concentrations of VOCs, spanning from 0 to 4125 ppm. Each concentration interval aligns with the sensor’s sensitivity, delineating clear and discernible data points across the graph. All curves exhibit linearity across the tested concentration range. Particularly noticeable is the response curve for acetic acid, which stands out by overlapping the y-axis at lower concentrations (as depicted in the Figure 9B inset), underscoring the sensor’s heightened sensitivity to acetic acid compared to the other tested VOCs. The slope of each curve acts as a sensitivity metric, further emphasizing the sensor’s strong affinity for the analyte.

Among the tested VOCs, FA emerges as the sole compound significantly impacting sensor responses, albeit at elevated concentrations.

**Figure 10:**
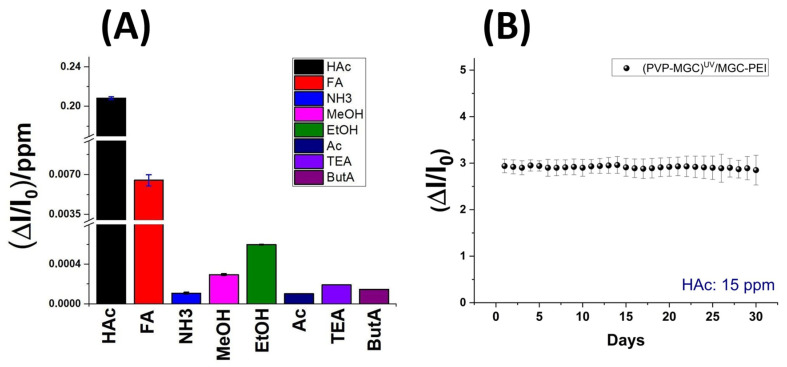
Sensitivity of the sensor towards each vapor solvent (**A**); sensor responses to 15 ppm HAc in 30 days (**B**).

The bar plot in Figure 10 depicts greater detail of the sensitivity values of the sensor to the tested VOCs. In order to be able to display all values, the y-axis was fragmented. The sensor exhibits minimal sensitivity to polar and small compounds like ethanol (EtOH) and methanol (MeOH). However, its sensitivity to amines, regardless of their structure (primary, secondary, or tertiary), and to ketones is negligible. This effect could be due to the mesoporous structure of the shell, which enables selective permeability. It could allow smaller molecules, such as HAc and FA, to diffuse through while excluding larger molecules.

Additionally, these mesopores may provide an extended surface area, enhancing their interaction with the functional groups and increasing adsorption. The presence of PEI in the shell, introducing amino groups able to form hydrogen bonds with the carbonyl groups of HAc and FA, could facilitate the selective adsorption of these carboxylic acids. The combined effects of the surface chemistry and pore structure result in increased sensitivity to acetic acid. Additionally, at higher concentrations, the enhanced diffusion of formic acid FA molecules through the shell leads to detectable levels of formic acid FA adsorption, expanding the detection capabilities of the sensor. Furthermore, the significant increase in current observed during the interaction between HAc molecules (acting as Lewis’s acids) and the MGC-PEI outer layer (with Lewis base sites) could result from the transfer of electrons from the Lewis base sites to the HAc molecules, thereby enhancing current flow. Additionally, the protonation of PEI molecules by acetic acid may modify the charge distribution within the composite, consequently increasing conductivity. An estimation of sensor selectivity [66] among the tested VOCs is calculated as follows:

$${Sel(A}_{k})=\left(\sum_{i=1}^{j}{S(A}_{k})/{S(A}_{i})\right)\times 100,$$

where *Sel* is the selectivity, S the sensitivity, and *A* is the analyte, which reveals that the sensor exhibits 96% sensitivity to acetic acid, 3% to formic acid, and 0.2% to ethanol. The other values are negligible.

The limit of detection (LOD), often defined as the concentration at which the signal-to-noise (S/N) ratio equals a 3 (LOD = 3 × Baseline Noise), represents the concentration at which the signal becomes three times higher than the baseline noise, ensuring reliable detection above the noise level. In our measurements, conducted up to 950 ppb, the LOD was determined to be 160 ppb (Figure 9A).

The sensor was tested for one month at the same concentration of acetic acid to evaluate its stability over time (Figure 10B). The response ((ΔI/I_0_)_mean_: 2.94 ± 0.15), averaged from five measurements per day, exhibited reproducibility ((ΔI/I_0_)_mean_: 2.91 ± 0.19) after 30 days of use. The sensors demonstrated a certain stability, with fluctuations remaining within the range of the measurement error.

Table 1 provides a comprehensive overview of the key sensing attributes of various nanostructured chemiresistors developed over the last decade, including the sensor developed in this study. It specifically compares sensor working temperatures and limits of detection, the key parameters affecting energy consumption and sensitivity, respectively.

Sensors operating at higher temperatures, despite their broad range of detection and fast responses, may not be optimal for wearable applications designed to monitor hazardous pollutants for workers. This is primarily due to their increased energy consumption, leading to a shortened battery life and necessitating more frequent recharging or replacement, which may not be practical for continuous use. Moreover, higher operating temperatures can expedite material degradation and necessitate additional thermal management systems to maintain stability and safeguard sensitive components. Consequently, the complexity and associated costs of thermal management diminish the appeal of sensors operating at higher temperatures for wearable devices.

Conversely, room-temperature chemosensors offer advantages such as simplicity, ease of use, and reduced energy consumption. However, they may encounter limitations related to sensitivity, selectivity, response time, susceptibility to environmental interference, and sensing material stability. To address these limitations, as elucidated in the Introduction paragraph, integrating graphene (RGO or G) with metals [32] or ceramics [33] has enabled chemiresistors to operate effectively at room temperature, achieving remarkable sensitivity with detection limits of up to 1 ppb [34].

The chemosensor developed in this study, consisting of electrospun PVP nanofibers filled with MGC and coated with PEI and MGC, demonstrates several competitive characteristics (Table 1). It exhibits sufficient sensitivity to detect low concentrations of the target analyte (LOD: 160 ppb) at room temperature, rendering it suitable for monitoring in various industrial or occupational settings. Moreover, the sensor displays high selectivity, with a specificity of 96% to acetic acid, enabling accurate discrimination of acetic acid from other gases or vapors in the environment, thereby minimizing false positives and ensuring precise detection and monitoring of acetic acid levels.

Furthermore, the chemosensor demonstrates strong stability over an extended period, maintaining consistent performance over 30 days of continuous measurements. This long-term stability ensures reliable and sustained operation of the sensor, reducing the need for frequent recalibration or maintenance. The fabrication process for the chemosensor is straightforward and easily implementable, making it accessible and cost-effective for large-scale production.

**Table 1:** A summary of recent research on gas sensors for the detection of acetic acid.

Sensing Material	Type of Sensor	Temperature (°C)	LOD	Reference
Pr-doped ZnO	Chemiresistor	380	50 ppm	[13]
Y-doped SnO_2_	Chemiresistor	300	10 ppm	[25]
Ag-doped LaFeO_3_	Chemiresistor	150	0.5 ppm	[67]
Flower-like SnO_2_	Chemiresistor	260	1 ppm	[24]
CdSxSe1−xnanoribbons	Chemiresistor	100	0.87 ppm	[68]
Gr:Au and Gr:Pt	Chemiresistor	rt	0.6%/ppm	[69]
Hexagonal ZnO	Chemiresistor	230	10 ppm	[16]
Tb_2_O_3_@MOF- ZnO	Chemiresistor	20 °C	0.5 ppm	[17]
Bi_2_O_2_CO_3_	Chemiresistor	150 °C	1 ppm	[70]
Co-doped SnO_2_	Chemiresistor	300 °C	10 ppm	[71]
metal oxide (WO/SnO)	Chemiresistor	rt	30 ppb	[18]
Sn_3_O_4_-RGO	Chemiresistor	rt	64%/ppm	[32]
C-doped α-Fe_2_O_3_	Chemiresistor	260 °C	1 ppm	[72]
Y-doped ZnO	Chemiresistor	350 °C	10 ppb	[12]
mesoporous CuO	Chemiresistor	200 °C	10 ppm	[31]
BaSnO_3_ microtubes	Chemiresistor	245 °C	0.3 ppm	[15]
ZnO foam	Chemiresistor	400 °C	0.5 ppm	[73]
GeC_3_N_4_ e SnO_2_	Chemiresistor	185 °C	0.1 ppm	[74]
MgGa_2_O_4_/graphene	Chemiresistor	rt	1 ppb	[34]
In_2_O_3_ nanofibers	Chemiresistor	250 °C	500 ppb	[21]
Mg-doped ZnO/rGO	Chemiresistor	250 °C	10 ppm	[33]
GQDs–ZnO	Chemiresistor	rt	1 ppm	[30]
PVP-MGC/MGC-PEI	Chemiresistor	rt	160 ppb	-

### 3.3. Portable Sensing System

Refocusing our attention on the human risks associated with exposure to hazardous concentrations of acetic acid, often challenging to measure in workplace environments, direct tests of this sensor using the previously described portable sensing system were carried out to assess its efficacy and practicality. As depicted earlier (Figure 2B), unlike the stationary system, where output is measured in current (A), the portable measurement system delivers output signals in voltage (V). The sensor’s interdigitated electrode (IDE) was connected to a dedicated electronic circuit board to ensure dependable measurements and housed within a Teflon measuring chamber installed on the board. Purified ambient air at 20 °C (±1 °C) and 45% relative humidity (±5%RH) was employed as the gas carrier, replicating realistic ambient conditions for these assessments. Each measurement was carried out for a duration of up to 90 s to ascertain if this timeframe allowed the sensor adequate time to detect the presence of acetic acid in the environment.

**Figure 11:**
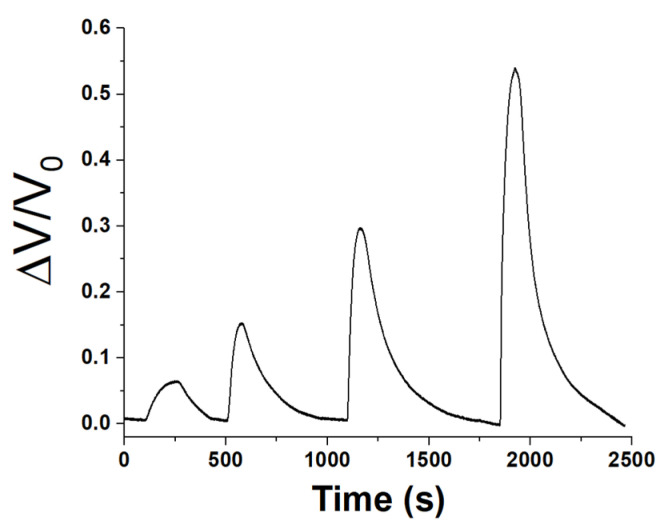
Normalized (ΔV/V_0_) response–recovery curves of the sensor when exposed to increasing concentrations of acetic acid vapors, ranging between 7 and 47 ppm.

Therefore, a sequence of progressively increasing exposure concentrations within 7 and 47 ppm, was generated and directed into the measurement chamber to evaluate the sensor’s linearity and sensitivity (refer to Figure 11).

After each exposure, the sensor was purged with purified ambient air only, facilitating the desorption of acetic acid from the sensor surface and restoring its original conductivity.

**Figure 12:**
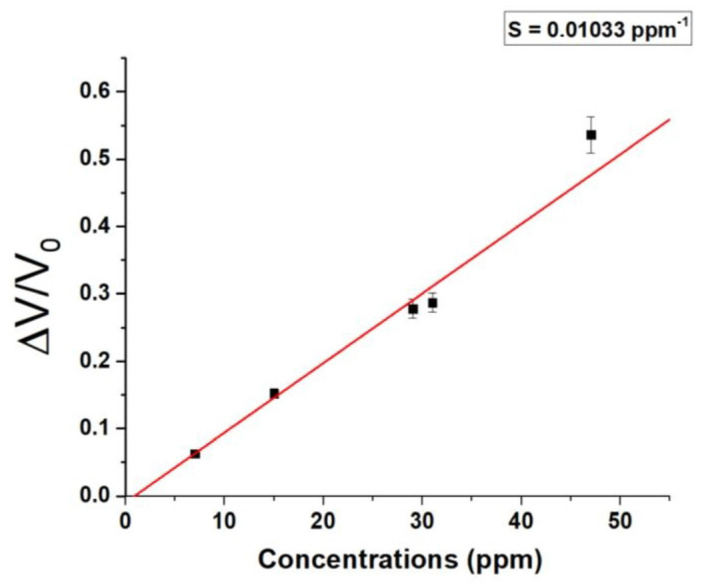
Normalized voltage responses (ΔV/V_0_) to different acetic acid vapors concentrations (ppm).

Based on the maximum signal attained, a calibration curve was derived (Figure 12).

The range encompasses all the concentrations under consideration, resulting in a linear fit curve (red) with a slope of 0.01033 ppm^−1^.

The integration of the sensor into a portable and low-cost system has reduced the sensitivity of the sensor, achieving a 1 ppm LOD and a 3 ppm LOQ (limit of quantification). LOQ represents the lowest concentration accurately quantifiable by the sensor with an acceptable level of precision and accuracy [75].

## 4. Conclusions

In this study, we explored the sensing properties of a fibrous mat comprising PVP-MGC decorated with PEI-MGC and deposited on an IDE. PVP demonstrates excellent spinnability, yielding uniform nanofibers with controlled morphology and structure. This uniformity allows homogeneous binding site distribution, contributing to the reliability and accuracy of the sensor. The UV curing of PVP nanofibers aims to ensure the long-term stability and performance of the sensor device, particularly for environmental applications. To enhance both electrical conductivity and sensing features, both the inner and outer layers of the nanofibers are functionalized with MGC nanopowder, serving dual roles as filler and surface binding agent.

The mesoporous structure of MGC provides abundant active sites for analyte adsorption, leading to enhanced sensing sensitivity. Additionally, the high conductivity of graphene facilitates efficient electron transfer, resulting in rapid and responsive sensing performance. The nanomaterial dimensions of the fibers, achieved through electrospinning technologies, provide a high surface area-to-volume ratio, further enhancing the material’s sensing properties.

Initial measurements revealed a notable sensing response of the nanofibers to carboxylic acids, particularly towards acetic acid vapors. The thin shell of polyethyleneimine (PEI) and mesoporous carbon graphitized (MGC) on nanocomposite fibers (PVP-MGC) provides an ideal platform for the adsorption and detection of acetic acid and a minor response towards the detection of formic acid. This behavior can be attributed to its surface chemistry, with a well-defined pore structure allowing selective permeability and increasing the sensitivity of the target analytes. The developed (PVP-MGC)^UV^/MGC-PEI structure is a combination of nanoarchitectures, strategies, and technologies validated by literature focused on acetic acid detection. However, in this work, we introduce an original composite material made of electrospun nanofibers of PVP doubly decorated with mesoporous graphene, offering distinct advantages over traditional sensing materials and facilitating detection capabilities. Finally, the synergistic effects of combining PVP nanofibers, MGC, and PEI created a multifunctional sensing platform with enhanced performance characteristics. This synergism led to improved detection limits, fast response times, and good stability compared to conventional sensing materials.

We tested two measurement systems: a stationary and a portable/low-cost system. Both systems demonstrated similar responses, with the stationary system exhibiting better accuracy offset compared to the portability of the latter. Here, the sensor looks to work stably and selectively at room temperature, detecting up to 160 ppb HAc (LOD) in ambient air with a selectivity of 96% among the tested VOCs. In contrast, the portable system can be directly used in workplaces, monitoring and storing data on workers’ exposure to acetic vapors. On the other hand, it displays a LOD of ~1 ppm and a LOQ of ~3 ppm, staying within workplace exposure limits based on TWA and STEL values. Based on this encouraging data, further developments could focus on enhancing the portable sensor unit for real-time monitoring, enabling timely alerts to workers in the event of dangerous concentrations, and facilitating wearable applications. The sensor’s design not only ensures rapid and reliable results but also opens up a world of possibilities for enhanced process control and risk mitigation in various industries. With its compact and cost-effective architecture, it may ensure the prevention of potential health risks, promoting a safer work environment.

The fabrication process for the chemosensor is straightforward and easily implementable, making it accessible and cost-effective for large-scale production. Additionally, its compact and lightweight construction enables seamless integration into wearable devices or personal protective equipment, facilitating continuous monitoring of acetic acid levels.

However, numerous factors still require definition to assess the potential usability of the sensor for prolonged use in relevant industrial or occupational settings. These factors include the impact of humidity and temperature changes on the sensor structure and functionality over time and potential hysteresis phenomena when exposed to high and prolonged concentrations of acetic acid (and/or interferents), among others.

In conclusion, while the presented study has made significant strides in the development and characterization of the sensor for detecting acetic acid vapors, it is important to acknowledge that further studies are necessary to fully evaluate its suitability for prolonged use in relevant industrial or occupational settings. Key factors such as the impact of environmental conditions, long-term stability, and potential hysteresis effects need to be thoroughly investigated. Additionally, ongoing research and collaboration with industry partners will be essential to address these challenges and optimize the sensor’s performance for occupational safety and environmental monitoring.

## Data Availability

All data that support the findings of this study are available after the reasonable request to the corresponding author.

## Supplementary Materials

The following supporting information can be downloaded at: https://www.mdpi.com/article/10.3390/s24072174/s1.
