# Supplementary material for: A Polyvinylpyrrolidone Nanofibrous Sensor Doubly Decorated with Mesoporous Graphene to Selectively Detect Acetic Acid Vapors
Source: Sensors (Basel). 2024 Mar 28;24(7):2174. doi: 10.3390/s24072174 (PMC11014041; doi:10.3390/s24072174)
Supplement: Supplementary file 1 [file sensors-24-02174-s001.zip › sensors-2920906-supplementary.pdf]

SUPPLEMENTARY MATERIAL TO THE ARTICLE SUBMITTED TO SENSORS (MDPI) AND TITLED:

A PVP nanofibrous sensor doubly decorated with mesoporous graphene to selectively detect acetic acid vapours

Paolo Papa , Emiliano Zampetti , Fabricio Molinari, Fabrizio De Cesare, Corrado Di Natale, Giovanna Tranfo and Antonella Macagnano

Electrospinning is a versatile and widely used technique for producing ultrafine fibers with diameters ranging from nanometers to micrometers. In this process, a high voltage is applied to a polymer solution or melt, typically contained in a syringe or reservoir. As the solution or melt is ejected through a spinneret, the electric field induces a charge on the surface of the droplet, causing it to elongate into a fine jet.

The repulsive forces between the charges overcome the surface tension of the solution or melt, resulting in the stretching and thinning of the jet as it travels towards a grounded collector. During flight, solvent evaporation or solidification occurs, leading to the formation of solid fibers that are collected on the grounded substrate or collector. The process parameters such as solution viscosity, flow rate, applied voltage, and distance between the spinneret and collector can be adjusted to control the morphology, diameter, and alignment of the electrospun fibers.

Electrospinning is widely used in various fields including tissue engineering, filtration, sensors, and drug delivery due to its ability to produce fibers with high surface area-to-volume ratio and tunable properties.

Here, the fiber deposition process utilized a Fluidnatek® LE-50 electrospinning machine (Bioinicia, Spain). To ensure the production of uniform and dry fibers, the distance between the needle and the collector was set at 9 cm, with a solution flow rate of 400 μL/h. The setup included two high-voltage sources: one at the needle with a voltage of +4.6 kV and the other at the collector with a voltage of -2 kV. A rotating cylinder collector (500 rpm) was employed to promote organized alignment of the fibers during deposition. Once the electric potential was applied, the polymeric dispersion jet coated the interdigitated electrodes (IDEs) secured to the collector using conductive tape and positioned inside the deposition cone.

Figure S1 provides a comprehensive overview of the electrospinning setup, demonstrating the key components and operational procedures involved in the deposition of nanofibers for sensor applications.

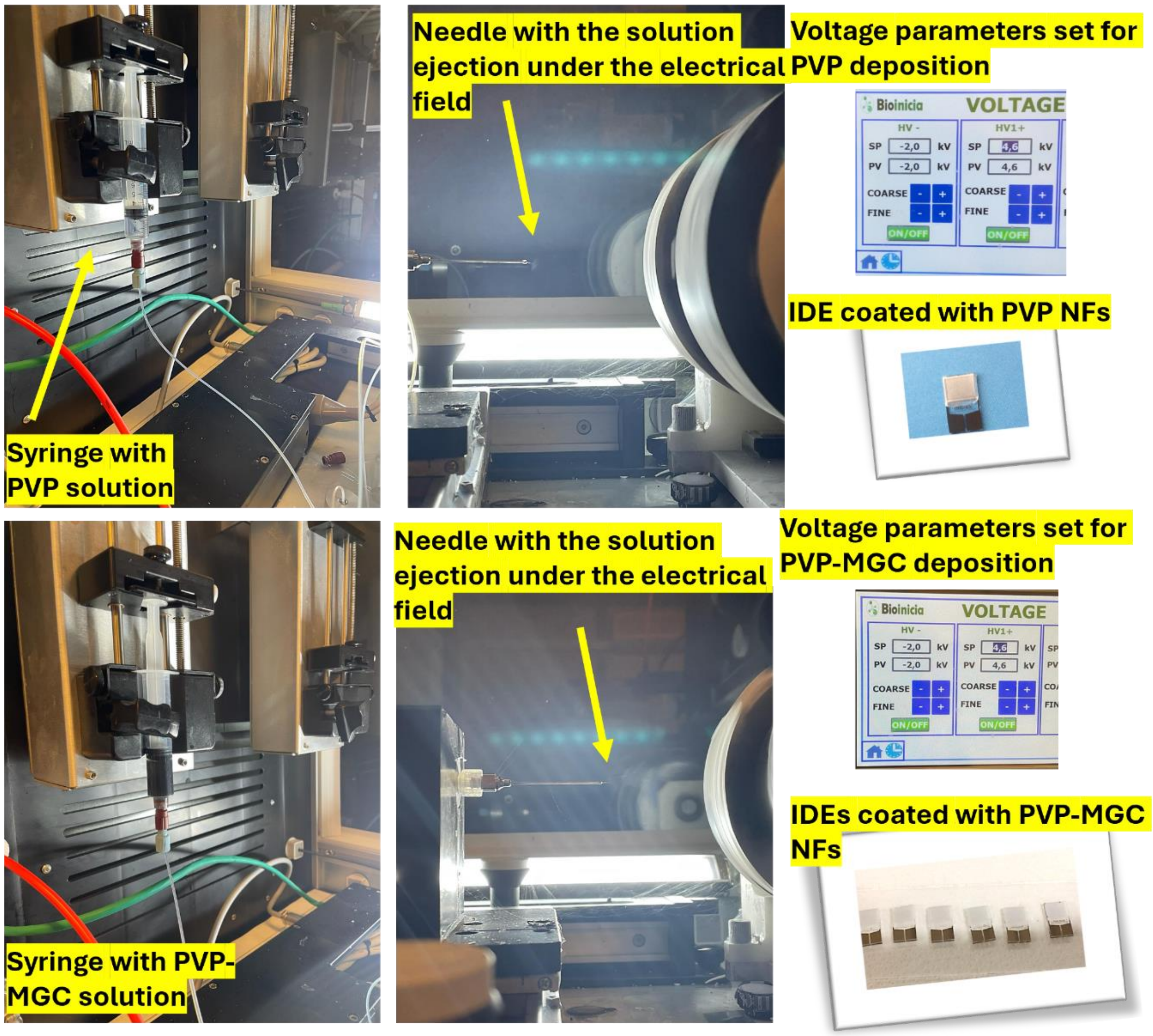
Syringe with PVP solution
Needle with the solution ejection under the electrical field
Voltage parameters set for PVP deposition
Bioinicia
VOLTAGE
HV -
HV1+
SP -2,0 kV
PV -2,0 kV
SP 4,6 kV
PV 4,6 kV
COARSE
FINE
ON/OFF
IDE coated with PVP NFs
Syringe with PVP-MGC solution
Needle with the solution ejection under the electrical field
Voltage parameters set for PVP-MGC deposition
Bioinicia
VOLTAGE
IDEs coated with PVP-MGC NFs
